# Trends in Urethral Suspension With Robotic Prostatectomy Procedures Following Medicare Payment Policy Changes

**DOI:** 10.1001/jamanetworkopen.2022.33636

**Published:** 2022-10-04

**Authors:** Jonathan Li, Dattatraya Patil, Benjamin J. Davies, Christopher P. Filson

**Affiliations:** 1 Department of Urology, Emory University School of Medicine, Atlanta, Georgia; 2 University of Pittsburgh School of Medicine, Pittsburgh, Pennsylvania; 3 Department of Urology, University of Pittsburgh Medical Center, Pittsburgh, Pennsylvania; 4 Winship Cancer Institute, Emory Healthcare, Atlanta, Georgia

## Abstract

**Importance:**

In 2016, the Centers for Medicare and Medicaid Services cut payments for robotic prostatectomy performed for Medicare beneficiaries. Although regulations mandate that billing for urethral suspension is only acceptable for preexisting urinary incontinence, reductions in reimbursement may incentivize billing for the use of this procedure in other scenarios.

**Objective:**

To assess trends and geographic variations in payments for urethral suspension with robotic prostatectomy in the context of Medicare payment policy.

**Design, Setting, and Participants:**

This US population-based retrospective cohort study analyzed data from the IBM MarketScan Commercial Claims and Encounters and Medicare Supplemental Database for men with employer-based insurance (primary commercial or Medicare supplemental coverage) who underwent robotic prostatectomy (*Current Procedural Terminology* [*CPT*] code 55866) between 2009 and 2019.

**Exposures:**

Time period and metropolitan statistical area of patient residence.

**Main Outcomes and Measures:**

Payment for urethral suspension (*CPT* code 51990) with robotic prostatectomy.

**Results:**

We identified 87 774 men with prostate cancer treated with robotic prostatectomy; 3352 (3.8%) had undergone urethral suspension. The mean (SD) patient age was 59.7 (6.5) years; 16 870 patients (19.2%) had Medicare supplemental coverage. From 2015 to 2016, median payments for robotic prostatectomy changed by −$358 (−17.0%) for Medicare beneficiaries vs −$9 (0%) for commercially insured patients. With urethral suspension vs without, median (IQR) episode payments for robotic prostatectomy were higher for commercially insured men ($3678 [$3090-$4503] vs $3322 [$2601-$4306]) and Medicare beneficiaries ($2927 [$2450-$3909] vs $2379 [$2014-$3512]). Compared with men treated between 2013 and 2015, those treated between 2016 and 2017 were twice as likely to undergo urethral suspension (8.5% vs 4.1%; odds ratio, 2.17 [95% CI, 1.96-2.38]). The proportion of patients who underwent urethral suspension was stable for 2018 to 2019 and 2016 to 2017 (8.5% vs 9.0%; odds ratio, 1.06 [95% CI, 0.96-1.18]). From 2015 to 2019, the proportion of patients who underwent urethral suspension was highest in Charleston, South Carolina (92.0%), Knoxville, Tennessee (66.0%), and Columbia, South Carolina (58.0%). These regions neighbored high-volume areas without patients who underwent prostatectomy with urethral suspension (eg, 146 patients in Greenville, South Carolina, and 173 in Nashville, Tennessee).

**Conclusions and Relevance:**

In this study, urethral suspension was associated with increased costs for patients with both commercial insurance and Medicare. Patients treated between 2016 and 2017 were more likely than those treated between 2013 and 2015 to undergo this procedure. Geographic variation in use exceeded what was expected for the preexisting condition for which billing is permitted for Medicare beneficiaries. Policy statements from professional societies highlighting appropriate billing for urethral suspension may have tempered, but not reversed, the broad adoption of this procedure.

## Key Points

**Question:**

Has billing for urethral suspension increased since the Centers for Medicare and Medicaid Services decreased reimbursement for robotic prostatectomy for Medicare beneficiaries in 2016, and are there geographic variations in billing for these procedures?

**Findings:**

In this cohort study of 87 774 men with prostate cancer and commercial or Medicare supplemental insurance, payments for urethral suspension with robotic prostatectomy were analyzed for 2009 to 2019. Payments for these procedures were more common after 2012, but there were no substantial changes following the 2016 Medicare payment cuts; in addition, billing varied widely across metropolitan areas, with some regions approaching universal use.

**Meaning:**

Despite Medicare policy precluding billing for urethral suspension to prevent urinary incontinence, this procedure was commonly completed with robotic prostatectomy between 2016 and 2019, particularly in some metropolitan regions.

## Teaser

This cohort study uses national commercial and Medicare supplemental insurance claims data to analyze trends in urethral suspension with robotic prostatectomy for men with prostate cancer after the Centers for Medicare and Medicaid Services reduced reimbursement for these procedures.

## Introduction

Each year, the US Centers for Medicare and Medicaid Services (CMS) evaluates the appropriateness of reimbursements for individual medical services delivered by physicians. Based on the estimated time and resources required as well as reimbursements for similar types of procedures, the CMS assigns work relative value units (wRVUs) for specific procedures in its annual Physician Fee Schedule. Within urology, fee adjustments have had varied effects on procedure use. For instance, reductions in reimbursement for androgen deprivation therapy injections (eg, leuprolide) have been associated with a significant decrease in inappropriate use among Medicare beneficiaries but not in appropriate use.^1^ In another example, an increase in professional fees for office-based (vs facility-based) cystoscopy was followed by a marked increase in the use of office-based procedures without an associated decrease in facility-based cystoscopy.^2^ However, reductions in physician reimbursement for intensity-modulated radiation therapy (IMRT) did not significantly affect its use for patients with prostate cancer treated at freestanding clinics.^3^

In October 2015, the CMS released the 2016 Medicare Physician Fee Schedule. This schedule reduced the wRVU for robotic-assisted laparoscopic radical prostatectomy by 33.4%—from 32.06 to 21.36. For physicians who perform prostatectomies and rely on wRVU-based compensation, this 33.4% wRVU reduction may motivate efforts to replace this decrease with adjunct procedures at the time of prostatectomy. For example, laparoscopic urethral suspension is an adjunct procedure that might be considered. Results from institutional cohort studies^4^ and a randomized trial^5^ suggest that anterior suspension of the urethra at the time of prostatectomy may accelerate the recovery of postoperative urinary continence. However, the CMS does not allow billing for preventive services unless permitted by a separate law or with a national coverage determination. Thus, if a patient undergoing prostatectomy does not have preexisting incontinence, a surgeon is not technically permitted to bill for preventive anterior urethral suspension. To this point, the American Urological Association (AUA) Coding and Reimbursement Committee released a policy brief in May 2017 stating that laparoscopic anterior urethral suspension should only be billed for patients with preexisting urinary incontinence.^6^

In this context, we aimed to characterize trends and geographic variations in payments for urethral suspension amid a background of Medicare reimbursement reductions for prostatectomy payments that went into effect in 2016. We hypothesized that the use of urethral suspension would be more common after the payment cuts and would vary considerably among metropolitan regions.

## Methods

### Data Source

We used the IBM MarketScan Commercial Claims and Encounters and Medicare Supplemental Database to identify the cohort for this study. This database contains enrollment data, claims for inpatient and outpatient care, and pharmaceutical claims from patients covered by employer-based commercial insurance or supplemental insurance for Medicare-eligible beneficiaries across the US. For Medicare-eligible patients included in this database, all billing claims related to Part A (inpatient) and Part B (outpatient) coverage are available.

Because this cohort study used deidentified data, the Emory University School of Medicine Institutional Review Board deemed it exempt from review; thus, informed consent was waived. The study followed the Strengthening the Reporting of Observational Studies in Epidemiology (STROBE) reporting guideline.

### Cohort

The study population included men with localized prostate cancer who underwent robotic-assisted laparoscopic radical prostatectomy (*Current Procedural Terminology* [*CPT*] code 55866) while covered by commercial (aged 40-64 years) or Medicare supplemental (aged ≥65 years) health insurance. To assess underlying comorbidity and preexisting urinary incontinence, we excluded patients aged younger than 40 years and those with a shorter insurance coverage period (<6 months) before surgery.

### Outcomes of Interest

Our primary outcome of interest was a payment for urethral suspension at the time of prostatectomy. This outcome was determined by whether a patient had a paid claim for urethral suspension and laparoscopic prostatectomy (*CPT* codes 51990 and 55866, respectively) during the same episode of care. To assess the context of other adjunct procedures with robotic prostatectomy, we also evaluated the proportion of patients who had undergone pelvic lymphadenectomy (*CPT* code 38571), which is consistently covered and not preventive in the setting of a cancer operation. We also assessed time trends in the proportion of patients who had 1 or more claims associated with a diagnosis of urinary incontinence before receipt of urethral suspension with robotic prostatectomy, which would be considered appropriate treatment based on CMS policy.

### Analysis of Payment Trends

To support our hypothesis that billing for urethral suspension would increase after the 2016 changes in Medicare reimbursement for robotic prostatectomy, we assessed the median payment for robotic prostatectomy (*CPT* code 55866) by year over the study time frame, stratified by insurance type (commercial vs Medicare supplemental). We also described median payments for all surgical procedures on the same day of service as the robotic prostatectomy (*CPT* codes 10021-69990) based on insurance type and whether urethral suspension was performed.

### Exposures of Interest

Our primary exposure of interest was time period as a 4-level variable: 2009 to 2012, 2013 to 2015 (before the new Medicare payment policy), 2016 to 2017 (before the AUA policy brief), and 2018 to 2019. The initial randomized trial supporting the use of urethral suspension at the time of robotic prostatectomy was published in 2012.^5^ With that background, we compared trends in receipt of urethral suspension and pelvic lymphadenectomy with robotic prostatectomy before (2012-2015) and after (2016-2019) the payment policy change.

Our secondary exposure of interest was geographic variation. We examined the proportion of patients who underwent robotic prostatectomy with urethral suspension across metropolitan areas, defined as US Census–defined metropolitan statistical areas (MSAs). Patients residing outside an MSA were considered together as a separate group. We limited this analysis to data from 2015 to 2019 and to MSAs where at least 40 robotic prostatectomies were performed.

### Statistical Analysis

We performed bivariate testing to evaluate the associations between receipt of urethral suspension and pertinent patient and geographic factors. These factors included patient age, health plan type, US Census region, and population of the metropolitan region. We created a multivariable logistic regression model to estimate the odds of receiving urethral suspension, with time period as the primary exposure. We included patient age (categorical), health plan type, US Census region, and population of the MSA of residence (categorical). We then performed interrupted time series analyses to evaluate trends in payments for urethral suspension and for pelvic lymphadenectomy at the time of robotic prostatectomy. We performed a difference-in-differences analysis of trends before and after the 2016 payment policy change according to insurance type (commercial vs Medicare supplemental).

Statistical significance was considered at the 5.0% level, and all tests were 2 sided. All analyses were performed using Stata 14/SE (StataCorp Inc) and SAS (SPSS Inc) software.

## Results

We identified 87 774 men treated with robotic prostatectomy. The mean (SD) patient age was 59.7 (6.5) years; 16 870 patients (19.2%) had Medicare supplemental insurance, and 70 904 (80.8%) had commercial insurance. The majority of the cohort (74 726 [85.1%]) resided within an MSA. Overall, 3352 men (3.8%) had a payment for urethral suspension associated with their prostatectomy. The associations between use of urethral suspension and patient factors are presented in eTable 1 in the Supplement. We noted more frequent use of urethral suspension for men with a high-deductible vs preferred or extended provider organization health plan (4.8% vs 3.7%; *P* < .001). Men who resided in the South were more likely to undergo urethral suspension compared with other regions (eg, 4.7% vs 2.8% in the West; *P* < .001). Men with commercial insurance comprised the majority (80.8%) of our cohort and were more likely to have undergone urethral suspension compared with those with Medicare supplemental coverage (4.0% vs 3.1%; *P* < .001). The proportion of men with preexisting incontinence who underwent urethral suspension remained stable at only around 20.0% to 30.0% over time (eFigure 1 in the Supplement).

**Figure 1.**
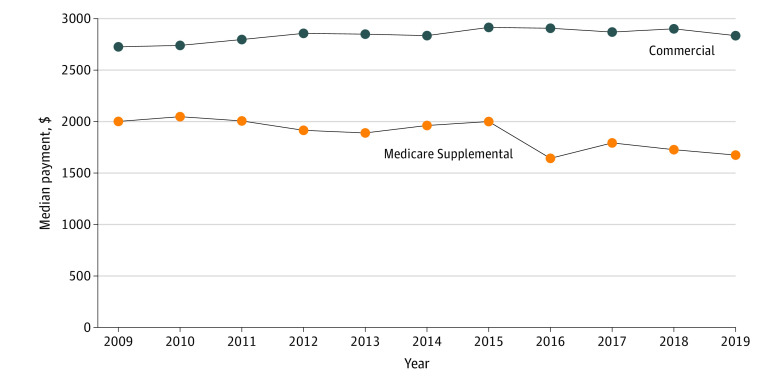
Annual Trends in Median Payments for Robotic Prostatectomy Based on Insurance Type (Commercial vs Medicare Supplemental) *Current Procedural Terminology* code 55866 was used for robotic prostatectomy.

We noted changes in payments for robotic prostatectomy after enactment of the 2016 CMS policy change (Figure 1). In 2009, payments for men with commercial insurance were considerably greater than those for patients with Medicare supplemental coverage ($2725 vs $2001). For Medicare-eligible men, median (IQR) payments for *CPT* code 55866 decreased 17.9% (from $2000 [$1745-$3211]) to $1643 [$1401-$3389]) between 2015 and 2016. Over the same period, payments for patients with commercial insurance decreased by $9 (0%). There were no substantial changes in payments from 2009 to 2019 for men with commercial insurance. Payments for urethral suspension vs no suspension increased overall median (IQR) surgical payments by 9.4% for commercially insured men ($3678 [$3090-$4503] vs $3322 [$2601-$4306]) and by 27.0% for Medicare beneficiaries ($2927 [$2450-$3909] vs $2379 [$2014-$3512]) (eFigure 2 in the Supplement).

**Figure 2.**
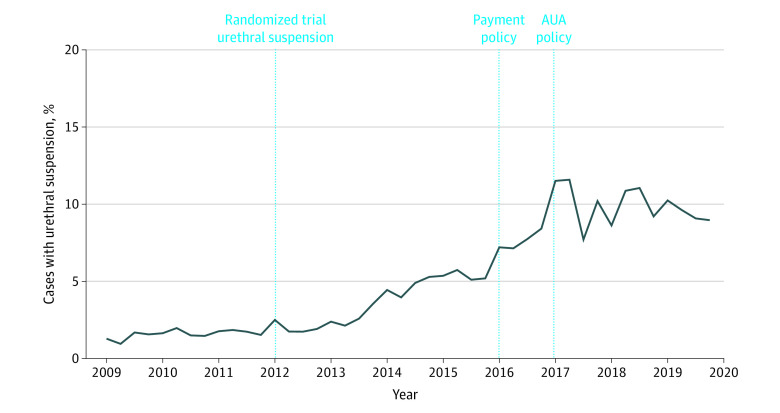
Proportion of Patients Undergoing Robotic Prostatectomy With Urethral Suspension in the Context of Pertinent Events, 2009 to 2019 AUA indicates American Urological Association.

**Figure 3.**
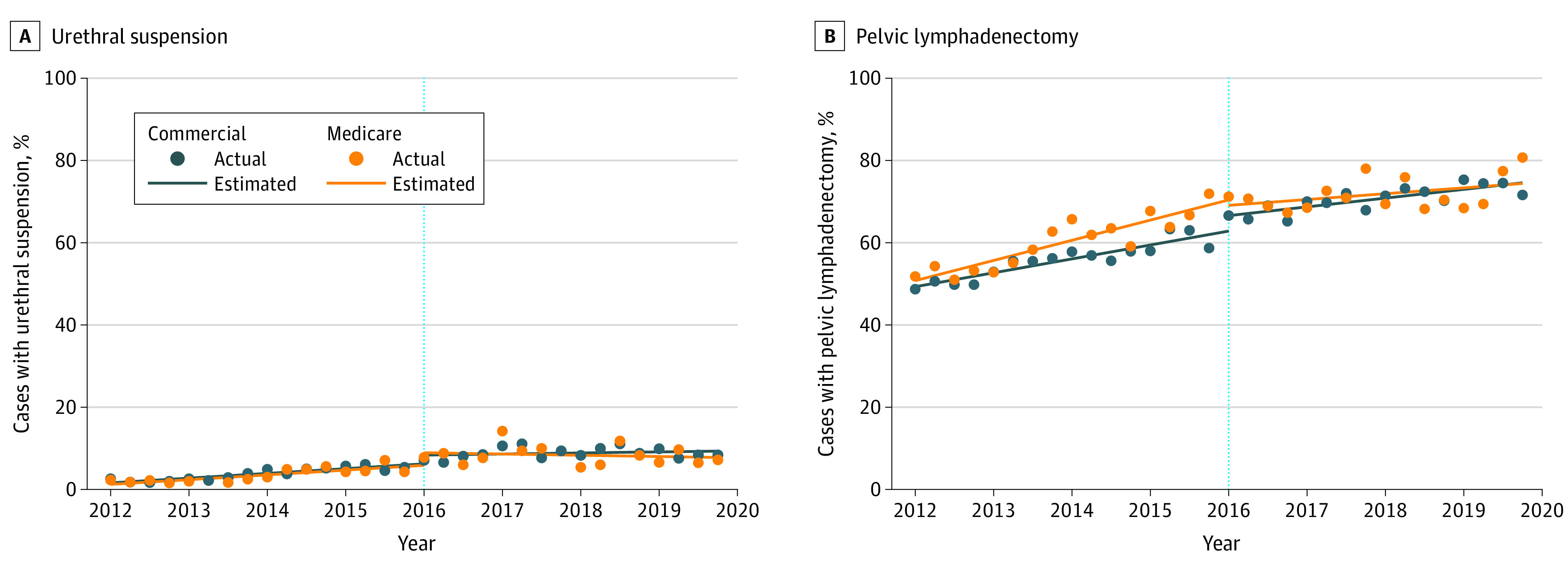
Interrupted Time-Series Analyses of Trends in Use of Urethral Suspension and Pelvic Lymphadenectomy at Time of Robotic Prostatectomy in the Context of the 2016 Medicare Payment Policy Change, 2012 to 2019 The dotted blue vertical lines indicate the date of Medicare payment policy change.

The proportion of patients who underwent prostatectomy with urethral suspension increased markedly over time from 1.7% in 2009 to 2012 to 9.0% in 2018 to 2019 (*P* < .001). Quarterly trends in the context of pertinent events are shown in Figure 2. Increased use of urethral suspension with prostatectomy began in 2012 and continued until 2017, after which it plateaued. After adjusting for other factors, men treated from 2013 to 2015 were less than half as likely to undergo adjunct urethral suspension as those treated from 2016 to 2017 (adjusted odds ratio [OR], 0.46 [95% CI, 0.42-0.51]). Men treated after 2017 were just as likely to undergo urethral suspension as those treated from 2016 to 2017 (8.5% vs 9.0%; adjusted OR, 1.06 [95% CI, 0.96-1.18]) (eTable 2 in the Supplement). Results from our interrupted time-series analyses for urethral suspension and pelvic lymphadenectomy are shown in Figure 3 and eTable 3 in the Supplement. Before 2016, urethral suspension use increased at the same rate of 0.3% per quarter for commercially insured patients (95% CI, 0.21%-0.37%) and Medicare beneficiaries (95% CI, 0.17%-0.41%). After 2016, use plateaued for both groups, with rates of 0.1% for commercially insured individuals (95% CI, −0.08% to 0.21%) vs −0.08% for Medicare beneficiaries (95% CI, −0.28% to 0.13%). There were no significant differences in change over time for patients with commercial insurance or Medicare supplemental coverage before (2012-2015) vs after (2016-2019) the policy change.

**Figure 4.**
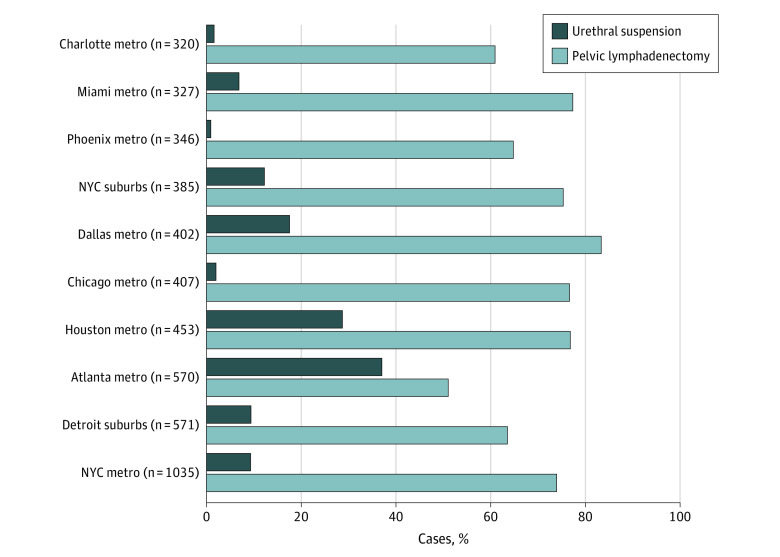
Variation in Use of Urethral Suspension and Pelvic Lymphadenectomy With Robotic Prostatectomy Across Metropolitan Statistical Areas (Metros) With the Highest Case Volume, 2015 to 2019 NYC indicates New York City.

Men in larger MSAs (>750 000 people) were more likely to undergo urethral suspension than those in smaller ones (<250 000 people) (4.2% vs 3.3%; adjusted OR, 1.23 [95% CI, 1.07-1.42]; *P* < .001) (eTables 2 and 3 in the Supplement). Figure 4 illustrates the percentages of urethral suspension and pelvic lymphadenectomy use among the 10 MSAs with the highest case volume from 2015 to 2019. We did not observe an association between performance of urethral suspension and pelvic lymphadenopathy. Notably, the Atlanta, Georgia, metropolitan area had the lowest proportion of patients receiving pelvic lymphadenectomy but the highest proportion undergoing urethral suspension. In other regions with at least 40 robotic prostatectomies performed between 2015 and 2019, the proportion of men undergoing urethral suspension was highest in the MSAs of Charleston, South Carolina (92.0%), Knoxville, Tennessee (66.0%), and Columbia, South Carolina (58.0%). These areas neighbored other regions without any payments for this adjunct procedure at the time of prostatectomy, including Greenville, South Carolina (146 patients), Augusta, Georgia (122 patients), and Nashville, Tennessee (173 patients). The highest-volume MSAs without any payments for urethral suspension included Minneapolis-St Paul, Minnesota (203 patients), Memphis, Tennessee (189 patients), and Kansas City, Missouri/Kansas (187 patients).

## Discussion

Two key findings from this cohort study are as follows. First, the adoption of urethral suspension at the time of robotic prostatectomy predated 2016 changes to Medicare reimbursement for prostatectomy and plateaued after a 2017 AUA policy brief recommended against billing for this adjunct procedure. Second, we observed a noticeable geographic variation in payments for urethral suspension between cities, with use rates for some MSAs that would far exceed the prevalence of preexisting incontinence for men considering robotic prostatectomy. These results may have policy implications for the potential spillover outcomes related to payment reductions for urologic oncology care.

In our analysis, we did not examine direct outcomes associated with Medicare payment policies related to robotic prostatectomy. Rather, we assessed indirect outcomes that surgeons may use to replace decreased reimbursement secondary to fee schedule changes with additional procedures performed at the time of prostatectomy (ie, urethral suspension). Mitchell et al^7^ described a similar spillover outcome among ophthalmologists who increased noncataract care for Medicare beneficiaries in response to reductions in fees for cataract-related care. Results from previous investigations of the outcomes of CMS payment policies related to urologic care delivery have been mixed. For example, Howard and Hockenberry^3^ observed that decreases in CMS payments targeted specifically for IMRT therapy in 2012 were not associated with reciprocal decreases in IMRT use overall, including for men with prostate cancer. Another institutional study reported a shift toward performing cystoscopy procedures in the office after the CMS increased fees for these procedures in 2005.^8^ A population-level analysis of Medicare beneficiaries with bladder cancer validated this finding and suggested that this policy did not just shift cystoscopy procedures between sites of care.^2^ Rather, care may have been expanded, as use of clinic-based procedures increased 644.0% after 2005 without similar decreases at facilities.^2^

In this study, we did not observe an association between the CMS policy change related to prostatectomy reimbursement and the use of urethral suspension. Increasing trends in the use of urethral suspension predated the policy change. There seems to have been a more marked transition in the quarterly trends after the AUA policy statement in 2017, which recommended against routine billing for urethral suspension at the time of prostatectomy. Although we cannot ascribe the changes observed in our cohort to that policy statement, there are other examples of clinical care outcomes at a population level with similar actions. Policy statements can have a marked effect on cancer care and screening, including use of screening mammography,^9^ prostate cancer screening,^10^ and prostate biopsies and prostate cancer treatment.^11^ Thus, we may have observed how the AUA policy statement contributed to slowing broad adoption of billing for urethral suspension at the time of prostatectomy.

Reporting geographic variation in surgical care is not new. However, we found that in some regions, payment for urethral suspension was more likely. Furthermore, some regions with frequent urethral suspension use had appreciably lower rates of pelvic lymphadenectomy at the time of robotic prostatectomy compared with other MSAs. This finding was particularly notable with our observation of many high-volume MSAs not having any payments for adjunct urethral suspension, including many in proximity to regions with heavy use. One possible explanation for this variation is that local payer coverage decisions may be more liberal in certain metropolitan regions. Another explanation is that the adoption of urethral suspension may be broader in certain areas in response to increased recognition of the benefits of this procedure for patients with preexisting urinary incontinence undergoing prostatectomy. In this hypothetical scenario, billing for this procedure is appropriate and perhaps expected to plateau at the level of prevalent urinary incontinence among this population. However, large cohort studies have suggested that the prevalence of preexisting urinary incontinence is almost negligible among patients undergoing robotic prostatectomy.^12^ We also noted that only approximately one-quarter of patients undergoing urethral suspension in our cohort had preexisting urinary incontinence. Geographic variation in prostate cancer care delivery is well established and has a number of motivating factors. For example, other work has highlighted variation in the use of risk stratification with prostate magnetic resonance imaging and genomic testing^13^ and active surveillance strategies for men with lower-risk prostate cancer.^14^ In this case, possible facilitators of this variation include a lack of knowledge of the AUA policy brief recommending against its use or even financial incentives promoting inappropriate use in the face of that policy recommendation.

### Limitations

Our findings should be viewed in the context of important limitations. The younger patients in our cohort were privately insured, and commercial payors likely did not adjust payments as quickly as the CMS did in 2016. However, it should be noted that 19.2% of patients in our cohort were covered under Medicare supplemental plans at the time of surgery. Second, the proportion of men who underwent urethral suspension was small relative to our population. Despite the relatively low frequency of our outcome, we detected a statistically significant association between our exposure and outcome. Third, we acknowledge that (1) the preventive exclusion policies stated by the CMS do not carry over explicitly to private payers and (2) professional policy statements simply represent guidance and do not have legal standing. Furthermore, assessment of the association of policy changes with the outcome of interest was limited by examining differences before and after a specific time point (ie, year of CMS policy change) without taking preexisting trends into account.

Understanding how CMS payment policy affects the delivery of surgical and cancer-related health care is crucial. Overall, surgical care comprises one-half of Medicare payments per year.^15^ Study limitations notwithstanding, our observations may help many stakeholders (eg, hospitals, large urology practices, and academic departments) anticipate the effect (or lack thereof) of CMS payment policy on reimbursements for and use of urologic surgery for patients with private insurance. Regarding urethral suspension specifically, definitive clarification of its value is needed. If urethral suspension is deemed important to help improve long-term quality of life after robotic prostatectomy, policy makers should advocate for legislation to allow for billing this service at the time of prostatectomy, even in the absence of preexisting incontinence. Even if the CMS eventually bundles anterior urethropexy into the prostatectomy procedure, that added effort would likely be reflected in its reimbursement across the board. Relevant to physicians, our findings may help surgeons appraise their own use relative to national trends. The downstream effects of the noted opportunities among stakeholders may translate into reduced health care costs and increased accessibility to beneficial surgical procedures for patients.

It is unclear how Medicare payment policies affected the use of adjunct urethral suspension for patients undergoing robotic prostatectomy for prostate cancer. Going forward, research in this area should focus on the explanatory factors behind region-to-region variability and patient-specific factors that may alter payments to surgeons. The balancing act of optimizing costs and improving outcomes remains an ever-elusive challenge that will require the input of all stakeholders and clinicians to eliminate wasteful spending while still ensuring quality care for all patients.

## Conclusions

In this study, urethral suspension was associated with increased costs for patients with both commercial insurance and Medicare. Patients treated between 2016 and 2017 were more likely than those treated between 2013 and 2015 to undergo urethral suspension, and statements from professional societies appeared to have slightly decreased its use starting in 2017 without fully reversing the increase.

## Notes

Supplement. **eFigure 1.** Proportion of Patients With a Diagnosis of Urinary Incontinence Before Radical Prostatectomy Based on Receipt of Urethral Suspension **eFigure 2.** Median Payments for Robotic Prostatectomy Episodes Based on Insurance Type and Use of Urethral Suspension **eTable 1.** Men Treated With Robotic Prostatectomy With or Without Payment for Urethral Suspension **eTable 2.** Estimates From the Multivariable Logistic Regression Model **eTable 3.** Results of Interrupted Time-Series Analyses
