# Supplementary material for: Trends in Urethral Suspension With Robotic Prostatectomy Procedures Following Medicare Payment Policy Changes
Source: JAMA Netw Open. 2022 Oct 4;5(10):e2233636. doi: 10.1001/jamanetworkopen.2022.33636 (PMC9533184; doi:10.1001/jamanetworkopen.2022.33636)
Supplement: Supplement. — eFigure 1. Proportion of Patients With a Diagnosis of Urinary Incontinence Before Radical Prostatectomy Based on Receipt of Urethral Suspension eFigure 2. Median Payments for Robotic Prostatectomy Episodes Based on Insurance Type and Use of Urethral Suspension eTable 1. Men Treated With Robotic Prostatectomy With or Without Payment for Urethral Suspension eTable 2. Estimates From the Multivariable Logistic Regression Model eTable 3. Results of Interrupted Time-Series Analyses [file jamanetwopen-e2233636-s001.pdf]

Supplementary Online Content

Li J, Patil D, Davies BJ, Filson CP. Trends in urethral suspension with robotic prostatectomy procedures following Medicare payment policy changes. *JAMA Netw Open*. 2022;5(10):e2233636. doi:10.1001/jamanetworkopen.2022.33636

eFigure 1. Proportion of Patients With a Diagnosis of Urinary Incontinence Before Radical Prostatectomy Based on Receipt of Urethral Suspension
eFigure 2. Median Payments for Robotic Prostatectomy Episodes Based on Insurance Type and Use of Urethral Suspension
eTable 1. Men Treated With Robotic Prostatectomy With or Without Payment for Urethral Suspension
eTable 2. Estimates From the Multivariable Logistic Regression Model
eTable 3. Results of Interrupted Time-Series Analyses

This supplementary material has been provided by the authors to give readers additional information about their work.

© 2022 Li J et al. *JAMA Network Open*.

eFigure 1. Proportion of Patients With a Diagnosis of Urinary Incontinence Before Radical Prostatectomy Based on Receipt of Urethral Suspension

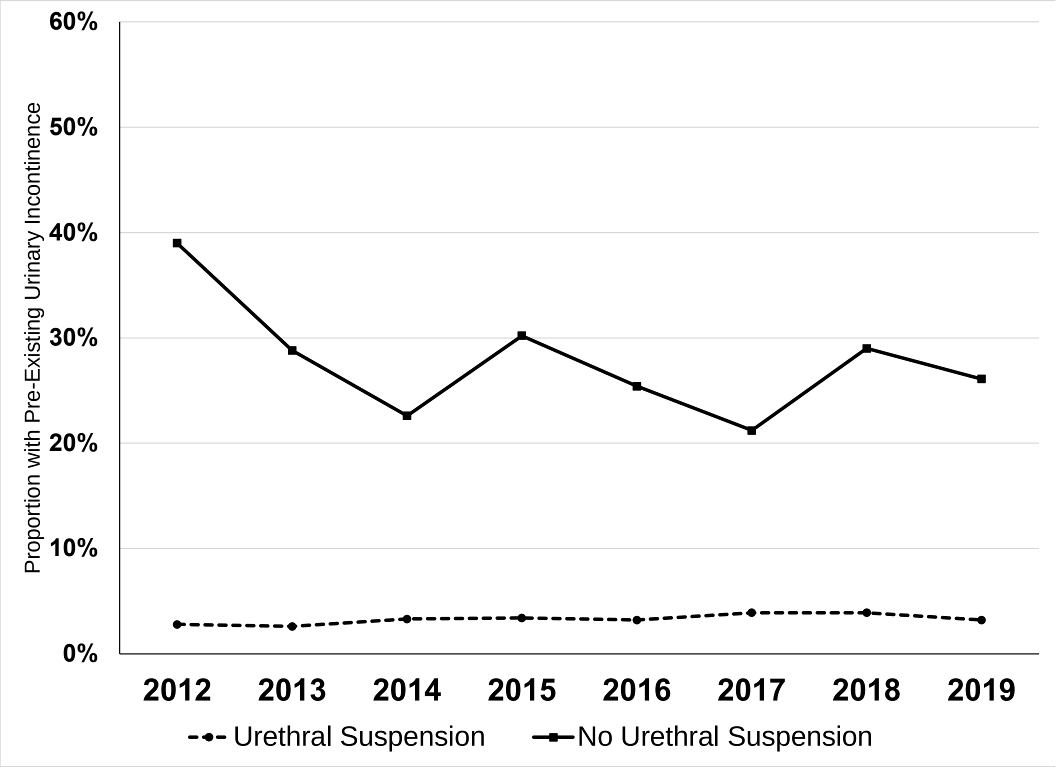

© 2022 Li J et al. *JAMA Network Open*.

eFigure 2. Median Payments for Robotic Prostatectomy Episodes Based on Insurance Type and Use of Urethral Suspension

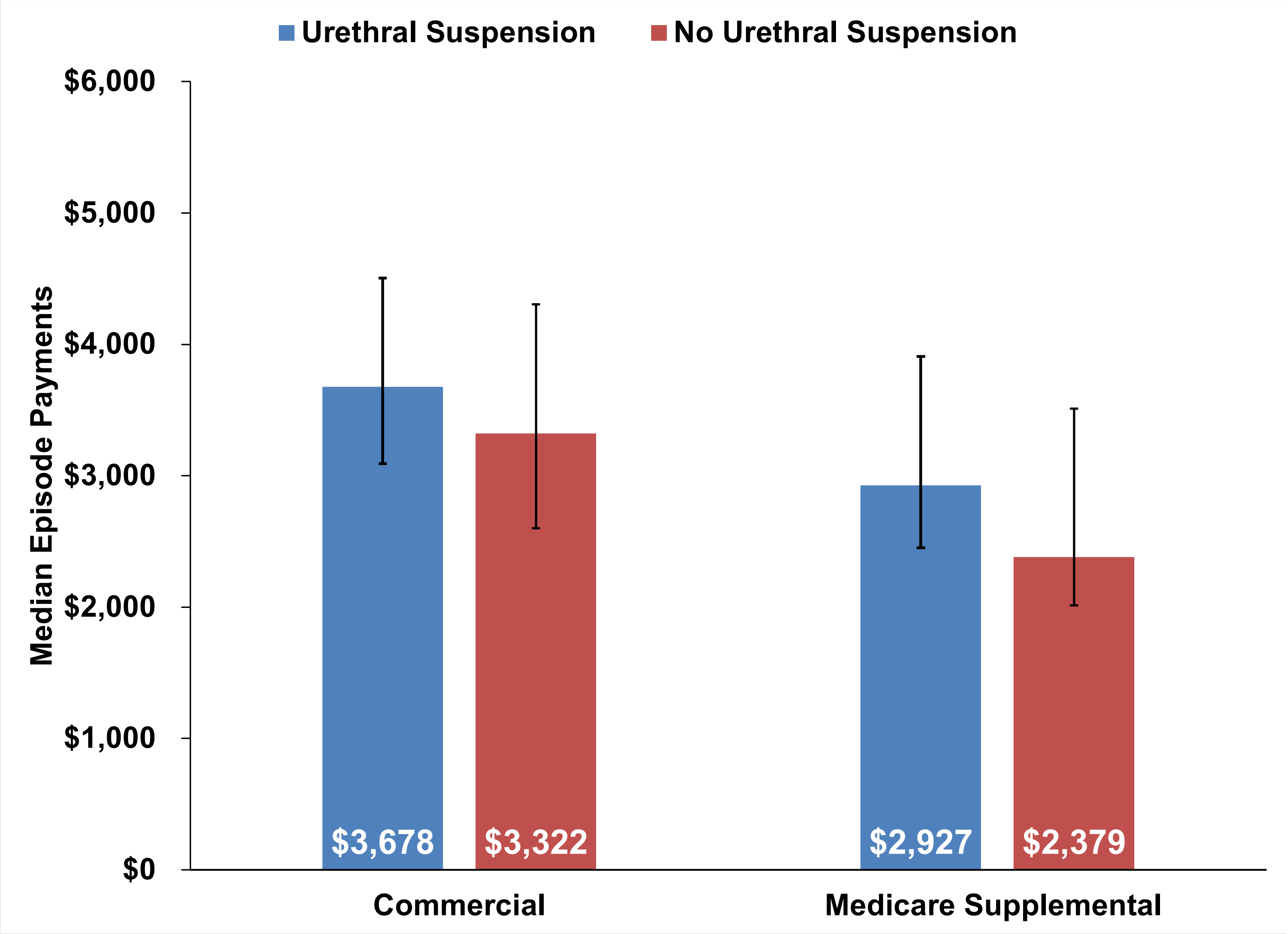

© 2022 Li J et al. *JAMA Network Open*.

eTable 1. Men Treated With Robotic Prostatectomy With or Without Payment for Urethral Suspension

Covariate	No Urethral Suspension (n = 84,422)	Urethral Suspension (n = 3,352)
Time Period		
2009–2012	45,559 (98.3)	793 (1.7)
2013–2015	21,821 (95.9)	929 (4.1)
2016–2017	9,412 (91.5)	878 (8.5)
2018–2019	7,630 (91.0)	752 (9.0)
Age (years)		
40–54	17,584 (96.4)	648 (3.6)
55–59	22,525 (96.0)	950 (4.0)
60–64	27,972 (95.8)	1,225 (4.2)
65–69	10,463 (96.9)	335 (3.1)
70+	5,878 (96.8)	194 (3.2)
Health Plan		
Comprehensive	6,740 (96.2)	265 (3.8)
HDHP/CDHP	8,152 (95.2)	411 (4.8)
HMO	8,377 (96.2)	331 (3.8)
POS/POS w/cap	6,060 (95.7)	271 (4.3)
PPO/EPO	51,033 (96.3)	1,976 (3.7)
Region		
Northeast	16,363 (96.0)	683 (4.0)
North Central	22,391 (97.0)	684 (3.0)
South	32,418 (95.3)	1,602 (4.7)
West	13,232 (97.2)	380 (2.8)
Unknown	18 (85.7)	3 (14.3)
Population of MSA (2015)		
Not in MSA	12,681 (97.2)	367 (2.8)
<250,000	6,941 (96.7)	237 (3.3)
250,000–749,999	14,856 (96.5)	540 (3.5)
750,000+	49,944 (95.8)	2,208 (4.2)
Payer Type		
Commercial	68,081 (96.0)	2,823 (4)
Medicare Supplemental	16,341 (96.9)	529 (3.1)

© 2022 Li J et al. *JAMA Network Open*.

eTable 2. Estimates From the Multivariable Logistic Regression Model

Covariate	OR (95% CI)
Year of surgery	
2009 – 2012	**0.19 (0.17-0.21)**
2013 – 2015	**0.46 (0.42-0.51)**
2016 – 2017	Reference
2018 – 2019	1.06 (0.96-1.18)
Health Plan	
HMO	Ref
Comprehensive	**1.25 (1.05-1.49)**
HDHP/CDHP	0.93 (0.80-1.08)
POS/POS w/ capitation	1.03 (0.88-1.22)
PPO/EPO	1.09 (0.97-1.23)
Region	
South	Ref
Northeast	**0.87 (0.79-0.96)**
North Central	**0.66 (0.60-0.72)**
West	**0.60 (0.53-0.67)**
Unknown	3.65 (1.08-12.35)
Population of MSA	
<250,000	Ref
250,000 – 749,999	1.05 (0.89-1.23)
750,000+	**1.25 (1.08-1.44)**
Non-MSA	**0.83 (0.70-0.99)**

© 2022 Li J et al. *JAMA Network Open*.

eTable 3. Results of Interrupted Time-Series Analyses

	Urethral Suspension				Pelvic Lymphadenectomy			
	2012 – 2015		2016 – 2019		2012 – 2015		2016 – 2019	
	Δ% per quarter	p	Δ% per quarter	p	Δ% per quarter	p	Δ% per quarter	p
Commercial	0.29 (0.21 – 0.37)	<0.001	0.06 (-0.08 – 0.21)	0.382	0.85 (0.62 – 1.07)	<0.001	0.53 (0.33 – 0.74)	<0.001
Medicare Supplemental	0.29 (0.17 – 0.41)	<0.001	-0.08 (-0.28 – 0.13)	0.452	1.23 (0.96 – 1.49)	<0.001	0.36 (-0.07 – 0.78)	0.099
Δ-Δ	0.00 (-0.15 – 0.14)	0.971	0.14 (-0.11 – 0.39)	0.264	-0.38 (-0.73 - -0.03)	0.032	0.18 (-0.30 – 0.65)	0.461

© 2022 Li J et al. *JAMA Network Open*.
